# How can continuing professional development better promote shared decision-making? Perspectives from an international collaboration

**DOI:** 10.1186/1748-5908-6-68

**Published:** 2011-07-05

**Authors:** France Légaré, Hilary Bekker, Sophie Desroches, Renée Drolet, Mary C Politi, Dawn Stacey, Francine Borduas, Francine M Cheater, Jacques Cornuz, Marie-France Coutu, Nora Ferdjaoui-Moumjid, Frances Griffiths, Martin Härter, André Jacques, Tanja Krones, Michel Labrecque, Claire Neely, Charo Rodriguez, Joan Sargeant, Janet S Schuerman, Mark D Sullivan

**Affiliations:** 1 Research Center of Centre Hospitalier Universitaire de Québec, Hospital St-François D'Assise, Québec City, Québec, Canada; 2 Leeds Institute of Health Sciences, School of Medicine, University of Leeds, Leeds, UK; 3 Department of Surgery, Division of Public Health Sciences, Washington University in St Louis School of Medicine, St. Louis, MO, USA; 4 School of Nursing, University of Ottawa, Ottawa, Ontario, Canada; 5 Continuing Professional Development Office, Faculty of Medicine, Université Laval, Québec City, Québec, Canada; 6 Institute for Applied Health Research, Glasgow Caledonian University, Glasgow, UK; 7 Department of Community Medicine, Centre Hospitalier Universitaire du Vaudois, Lausanne, Switzerland; 8 Centre for Action in Work Disability Prevention and Rehabilitation, School of Rehabilitation, Université de Sherbrooke, Longueuil, Québec, Canada; 9 Lyon 1 University, GATE-LSE (UMR 5824 CNRS), Lyon, France; 10 Health Sciences Research Institute, University of Warwick, Coventry, UK; 11 Institute and Policlinic for Medical Psychology, Center for Psychosocial Medicine, University Medical Center Eppendorf, Hamburg, Germany; 12 Practice Enhancement Division, Collège des médecins du Québec, Montreal, Québec, Canada; 13 Institute of Biomedical Ethics, University of Zurich, Zurich, Switzerland; 14 Institute for Clinical Systems Improvement (ICSI), Bloomington, MN, USA; 15 Department of Family Medicine, McGill University, Montreal, Québec, Canada; 16 Continuing Medical Education, Faculty of Medicine, Dalhousie University, Halifax, Nova Scotia, Canada; 17 Department of Psychiatry and Behavioral Sciences, University of Washington, Seattle, WA, USA

## Abstract

**Background:**

Shared decision-making is not widely implemented in healthcare. We aimed to set a research agenda about promoting shared decision-making through continuing professional development.

**Methods:**

Thirty-six participants met for two days.

**Results:**

Participants suggested ways to improve an environmental scan that had inventoried 53 shared decision-making training programs from 14 countries. Their proposed research agenda included reaching an international consensus on shared decision-making competencies and creating a framework for accrediting continuing professional development initiatives in shared decision-making.

**Conclusions:**

Variability in shared decision-making training programs showcases the need for quality assurance frameworks.

## Introduction

Shared decision-making (SDM) is an interactive process during which patients and practitioners collaborate in choosing healthcare. SDM is the crux of patient-centered care [1]. SDM is achieved when both patients and providers understand the best available evidence on the risks and benefits of available options and choose a course of treatment that takes patients' values and preferences into account [2-4]. For a number of reasons (fostering the use of evidence, respecting patient autonomy, etc.), stakeholders' preferred mode for clinical decision making is shifting from a paternalistic model to a model consistent with SDM [5]. A significant proportion of patients prefer to take an active role in decisions concerning their health, especially once they understand the benefits of doing so [6]. For example, patients' participation in decision making is associated with favorable health outcomes [7,8]. Moreover, interest in patients' active participation in medical education is also increasing [9,10].

Continuing professional development (CPD) is an important means by which health professionals keep abreast of the latest advances in healthcare [11]. Given the importance of healthcare professional training to the implementation of SDM in clinical practice, our international collaboration sought to increase the knowledge base of CPD programs and activities that seek to translate SDM into clinical practice, especially in primary care [12].

As planned in our protocol [12], we organized a two-day workshop in Quebec City, Canada, in November 2010. The principal investigator personally invited 35 individuals and one moderator to attend. Participants came from six countries (Canada, France, Germany, Switzerland, the United Kingdom, and the United States) and represented seven disciplines. There were 14 health services researchers [12-26], 11 trainees (five master's degree students, four postdoctoral fellows, and two PhD candidates), 5 research professionals, 3 CPD managers, and 2 representatives of a large healthcare organization. The objectives of the workshop were (a) to discuss participants' knowledge and perspectives on using CPD activities to cause SDM to be practiced in primary care, (b) to review the preliminary results of the environmental scan, (c) to use the preliminary results to identify knowledge gaps, and (d) to set a research agenda.

The workshop had two main components (see Additional File 1). The first component consisted of the following elements: two keynote presentations; country presentations where participants synthesized their country's experience with implementing SDM in clinical practice, especially as concerned training health professionals in SDM; and the presentation and discussion of the preliminary results of an environmental scan. Additional File 2 presents the list of speakers.

The second component of the workshop consisted of two small group discussions and two plenary sessions. The workshop concluded with participants drawing on the group discussions and the previous day's presentations to construct a research agenda.

### Keynote presentations

In the first keynote presentation, two representatives from the Institute for Clinical Systems Improvement summarized how to develop a program to train health professionals in SDM. Comprised of 60 medical groups representing 9,000 physicians [27], the Institute uses collaborative and innovative processes to unite stakeholders around transforming healthcare systems to deliver patient-centered, evidence-based care. The Institute's objectives are to encourage the delivery of care that is consistent with the values and preferences of patients and families, to increase SDM, to augment patients' satisfaction with the decision-making process, and to encourage the appropriate use of resources.

In the second keynote presentation, Joan Sargeant of Dalhousie University, Canada, described how to use multisource feedback to assess physicians' performance and suggest improvements [28]. Sargeant also discussed the evaluation of outcomes of educational programs based on the Kirkpatrick evaluation framework for CPD [29] and the CPD accreditation standards of the Association of Faculties of Medicine of Canada [30].

### Country presentations

Representatives from each country summarized the state of SDM in their country, spoke of challenges to implementing SDM in clinical practice, and reviewed SDM training activities for healthcare professionals. Representatives from Canada, Germany, the United Kingdom, and the United States described CPD programs, and representatives from France and Switzerland described training in development. Representatives from Canada and Germany reported on the evaluation of training programs, and national policy or laws concerning SDM were presented for France, Germany, Switzerland, the United Kingdom, and the United States. One example of a policy was the National Health Service (NHS) of the United Kingdom's recent stipulation that SDM would become the norm--the "No decision about me without me" campaign [31]. The NHS will also pay providers for their performance, so that payment reflects outcomes, not just activity, and incentivizes medical staff to provide better care. Another policy example was France's 2002 law to protect patients' rights to information and hold physicians accountable for fully informing their patients so that individuals can make their own health decisions, based on information and advice supplied by their healthcare provider.

Representatives of Canada, Germany, Switzerland, and the United States explained particularities regarding SDM in their healthcare systems. In the United Kingdom, for example, physicians deliver care based on evidence of effectiveness, including cost effectiveness, while in Switzerland, the pharmaceutical industry wields substantial influence at all levels. Some representatives spoke of barriers to the practice of SDM: In the United States, implementing SDM is made more difficult by the fact that healthcare is delivered by independent groups. Participants also reported on the implementation of decision aids, on prevention and health-promotion measures, and on current research initiatives for developing and implementing SDM. Participants reported more training activities in SDM for health professionals since 2007 [32-36] and the appearance of SDM on the policy agenda of more countries (*e.g.*, Switzerland).

### Preliminary results of the environmental scan

Our aim for the meeting was to present the preliminary results of the scan and to explore how to improve and complete this part of our protocol [12]. Briefly, we relied on three main sources of data: (1) members of our team and their networks, (2) organizations and individuals involved in training healthcare professionals, and (3) systematic reviews in SDM. We sought out any CPD activity or CPD program (*i.e.*, set of activities), published or unpublished, in whatever language, that targeted SDM. Because our search strategy favored sensitivity over specificity, we considered all SDM training programs, including those in clinical settings other than primary care. We also included stand-alone activities when the full program material was not available to us. Once we had identified the programs, reviewers extracted each program's main characteristics: the program name, the nature of the material available and extracted, author contact information, the creation or publication date, the country of origin, and the languages in which the program was available. We also extracted information about the programs' educational features: their conceptual underpinnings; the rationale for developing the program; the sources that informed the program; the healthcare professionals targeted; the clinical context; the program's objectives and duration; its components and activities (*e.g.*, small group discussion, case study, role play, simulation); essential elements of SDM covered by the program [37]; and information about how the program was assessed, including the levels of assessment (*i.e.*, participants' reaction, their degree of learning, changes in their behavior, and changes in patient outcomes) [29].

At the group meeting, the team explained the data-extraction process and presented its findings: detailed information about 53 programs from 14 countries, published in 9 languages. Because six programs were identified late, the team only extracted the data from 47 programs. Of these, 34 programs targeted licensed health professionals and were retained as CPD programs. The clients of those programs were mostly physicians (n = 34) and/or nurses (n = 13). Most programs mentioned primary care (n = 37). There was considerable heterogeneity in the programs' duration (three hours or less to more than three days) and in their teaching methods, which included large group sessions (n = 32), small group sessions (n = 25), auto-tutorials (n = 15), the dissemination of printed educational material (n = 16), audit and feedback (n = 13), case discussions (n = 26), simulations (n = 23), and self-evaluations (n = 12). More programs took place in cancer (n = 7) than in any other clinical area, but cardiovascular diseases, diabetes, chronic pain, prenatal screening, and other areas were represented as well. Most programs covered the nine essential elements of SDM identified in the integrated SDM model developed by Makoul and Clayman (2006). We also discussed an important limitation of our scan; namely, that we included SDM training programs and stand-alone activities independent of the formats in which they were available to us (although we asked authors for all materials used in their programs, we did not always obtain it). This meant that we extracted data from diverse formats (*e.g.*, a PowerPoint presentation, a class syllabus, trainer and trainee manuals, a DVD, an auto-tutorial), which made it difficult to compare programs, since the information contained in a PowerPoint presentation, for example, is not as extensive as that contained in a trainer's manual. We specified to workshop participants that each program had only been extracted by one person and that we had not assessed the programs' quality but that we intended to use workshop participants' feedback to improve the scanning process after the workshop. We also mentioned that in future work of this genre, we intended to solicit the feedback of patient representatives as well as that of academics and managers.

### Group discussion

We held two small group discussions. For each one, we divided the participants into four groups of eight people from diverse backgrounds. In each group, one person took detailed notes and a second reported back during the plenary sessions. Additional File 3 details the discussion questions and their main outcomes. Briefly, participants requested a detailed list of the SDM-CPD programs covered by the scan, as well as a list of programs that had been excluded, together with the reasons for their exclusion, with a view to verifying whether programs were missing. They suggested ways to improve reporting (*e.g.*, scoring programs' success at identifying success factors and best practices, such as the most effective time frames). They found that the SDM-CPD programs identified in the scan showed great variety and suggested pursuing the search for programs or performing a systematic review instead of an environmental scan. They also suggested extracting more information about the programs, such as the type of conceptual model used (an SDM model or an educational model); stating whether the program hailed from a unit devoted to SDM or patient participation or a similar topic, or whether it came under the umbrella of a more generic CPD institution; and specifying the types of learning activities practiced (oriented around skills, attitudes, or knowledge) and each program's core objectives. They also asked that the programs be appraised in light of accreditation standards and suggested asking the program developers to validate the data extracted from their program (member checking). They suggested performing subgroup analysis (based on country or clinical area, for example) or focusing solely on postlicensure programs (*i.e.*, CPD).

With regard to the research agenda, citing the International Patient Decision Aid Standards research group [38-41], participants suggested building international consensus on a core set of competencies for SDM; these competencies, together with CPD accreditation standards, could then be drawn upon to develop certification criteria for SDM-CPD programs.

### Workshop evaluation

At the end of the workshop, we collected 18 evaluation sheets. The mean scores for the 10 items (range of 1 = not at all to 5 = definitively) ranged from 4.3 (for "Were the presentations scientifically balanced?") to 4.8 (for "Were the discussion sessions useful?" and "Did the speakers and moderator encourage the audience's involvement?"). Participants also identified weaknesses, proposed improvements, and suggested next steps (see Additional File 4).

## Conclusion

To the best of our knowledge, this meeting was the first to discuss an inventory of SDM-CPD programs across health professions and countries and the first to reach consensus on a detailed research agenda. Our next step will be to finalize the environmental scan based on participants' feedback. We will also work with a larger pool of stakeholders to (a) explore the feasibility and acceptability of participants' suggestion to establish an international consensus on core SDM competencies for SDM-CPD programs, (b) seek consensus on ways to evaluate CPD interventions, (c) create an evaluation framework based on accreditation standards, and (d) construct a grid or checklist for accrediting SDM-CPD programs based on the framework mentioned. Finally, we will consider applying for grants to develop and pilot SDM-CPD programs across healthcare professions and countries.

## Competing interests

The authors declare that they have no competing interests.

## Authors' contributions

All authors have made substantial contributions to the conception and design of this study and to the acquisition of data for the environmental scan. All authors attended the workshop. FL and RD drafted the manuscript. All authors revised it critically for important intellectual content and all approved the final version submitted.

## Supplementary Material

Additional file 1 Appendix 1. Workshop Agenda Click here for file

Additional file 2 Appendix 2. List of Speakers and Participants Click here for file

Additional file 3 Appendix 3. Questions and Answers for the Small Group Discussions Click here for file

Additional file 4 Appendix 4. Workshop Evaluation Click here for file
