# Supplementary material for: How can continuing professional development better promote shared decision-making? Perspectives from an international collaboration
Source: Implement Sci. 2011 Jul 5;6:68. doi: 10.1186/1748-5908-6-68 (PMC3154854; doi:10.1186/1748-5908-6-68)
Supplement: Additional file 1 — Appendix 1. Workshop Agenda [file 1748-5908-6-68-S1.DOC]

**Appendix 1: Workshop Agenda**

| Day 1 | | |
| --- | --- | --- |
| 12:00 | 13:00 | Registration |
| 13:00 | 13:15 | Welcome and housekeeping items ( France Légaré and moderator Marc Charland) |
| 13:15 | 13:45 | *Clinician's Perspective: Collaborative Conversations Support SDM*  Janet Schuerman and Dr Claire Neely, Institute for Clinical Systems Improvement |
| 13:45 | 14:00 | Questions and comments |
| 14:00 | 14:45 | Presentations by representatives from   - Germany: Martin Härter - France: Nora Moumjid - The United States: Mary Politi and Mark Sullivan   Each country has 15 minutes: 10 minutes for its presentation and 5 minutes for questions. |
| 14:45 | 15:15 | Break |
| 15:15 | 16:00 | Presentations by representatives from   - The United Kingdom: Hilary Bekker, Francine Cheater and Frances Griffiths - Switzerland: Jacques Cornuz and Tanja Krones - Canada : Dawn Stacey, Sophie Desroches and Marie-France Coutu   Each country has 15 minutes: 10 minutes for its presentation and 5 minutes for questions. |
| 16:00 | 16:30 | *Environmental scan: Flow trial and data extraction synthesis – preliminary results*  France Légaré and Renée Drolet, Research Center of the Centre Hospitalier Universitaire de Québec |
| 16:30 | 16:45 | Questions and comments |
| 16:45 | 17:00 | Wrap-up: moderator |

| Day 2 | | |
| --- | --- | --- |
| 07:00 | 08:30 | Continental breakfast |
| 08:30 | 08:45 | Greetings, brief review of Day 1 and housekeeping items: F Légaré and moderator |
| 08:45 | 09:15 | *The patient perspective: Views from multi-source feedback and CPD research*  Joan Sargeant, CPD director and researcher, Dalhousie University |
| 09:15 | 09:30 | Questions and comments |
| 09:30 | 09:35 | Introduction of small group discussions (questions 1.1 and 1.2) |
| 09:35 | 10:35 | Small group discussions: 4 groups of 8 people each |
| 10:35 | 11:00 | Break |
| 11:00 | 12:00 | Plenary: Each group presents a brief summary of its discussion. |
| 12:15 | 13:30 | Lunch |
| 13:30 | 13:35 | Introduction of small group discussions (questions 2.1, 2.2. and 2.3) |
| 13:35 | 14:35 | Small group discussions: 4 groups of 8 people each |
| 14:35 | 15:00 | Break |
| 15:00 | 16:00 | Plenary: Each group presents a brief summary of its discussion. |
| 16:00 | 16:30 | Next steps and closing remarks |

Notes: CPD=continuing professional education

SDM=shared decision making
