# Supplementary material for: How can continuing professional development better promote shared decision-making? Perspectives from an international collaboration
Source: Implement Sci. 2011 Jul 5;6:68. doi: 10.1186/1748-5908-6-68 (PMC3154854; doi:10.1186/1748-5908-6-68)
Supplement: Additional file 2 — Appendix 2. List of Speakers and Participants [file 1748-5908-6-68-S2.DOC]

Appendix 2: List of Speakers and Participants

|  | | | **Keynote Presentations** | | |
| --- | --- | --- | --- | --- | --- |
| **Presenters** | **Affiliation** | **Professional activity** | | **Research Interests** | **Title of presentation** |
| Janet Schuerman and Claire Neely | Institute for Clinical System Improvement (ICSI), Bloomington, MN, USA | SDM project leader and Medical director at ICSI | | - SDM | Clinician's Perspective: Collaborative Conversations Support SDM |
| Joan Sargeant | Dalhousie University | Director research and evaluation office of CME | | - CME - Knowledge translation - Communication skills - Performance assessment, feedback, practice improvement and self-assessment. | The patient perspective: Views from multi-source feedback and CPD research |
|  | | | **Presentations by Country Representatives** | | |
| **Presenters** | **Affiliation** | **Professional activity** | | **Research Interests** | **Country** |
| Martin Härter | Universitätsklinikum Hamburg-Eppendorf | Director of the Institute and Policlinic of Medical psychology | | - Patient involvement and SDM - Psycho-oncology and psycho-cardiology - Innovative care models for mental disorders - Health services research | Germany |
| Nora Moumjid | Claude Bernard University Lyon 1 | Assistant professor in health economics at the faculty of pharmacy | | - SDM - Decision aids - Healthcare users ‘elicitation preferences - Contingent evaluation - Risk communication | France |
| Mark Sullivan | University of Washington | Professor in psychiatry and behavioural sciences  Adjunct professor in bioethics and humanities | | - Chronic non-cancer pain - SDM - Collaborative care - Depression Care - Palliative care - Self-management | United States |
| Mary Politi | Washington University in St. Louis | Assistant professor | | - SDM - Patient-clinician communication - Health communication - Cancer prevention and control | United States |
| Hilary Bekker | University of Leeds | Senior lecturer (Associate Professor) in behavioural Sciences | | - Decision aid - Informed decision making - Using theory to understand and facilitate patient and professional decision making | United Kingdom |
| Jacques Cornuz | University of Lausanne | Associate Professor | | - Preventive medicine - General medicine - Clinical epidemiology - Smoking cessation - Medical education - Evidence-based medicine | Switzerland |
| Dawn Stacey | University of Ottawa | Associate professor, School of Nursing  Director, patient decision aids research group | | - Decision support - Decision coaching - SDM - Telephone care - Knowledge translation - Implementation research | Canada |
| Tanja Krones | University of Zurich | Clinical Ethicist/Executive of the Clinical Ethics Committee of the University Hospital and University of Zurich | | - SDM - Polimorbidity and SDM/Ethics | Switzerland |
| Other participants | | | | | |
|  | | | | | |
|  | Disciplines/occupation | | | Affiliation | County |
| N=4 | Health Services researchers | | | - Laval Université - Sherbrooke University | Canada |
| N=2 | Health Services researchers | | | - Glasgow Caledonian Université - University of Warwick | UK |
| N=2 | CPD managers | | | - Collège des médecins du Québec - Laval University | Canada |
| N=2 | Post-doctoral fellows | | | - Laval University - Ottawa University | Canada |
| N=2 | Post-doctoral fellows | | | - Mayo Clinic - University of Michigan | USA |
| N=2 | PhD candidate | | | - McGill University - Sherbrooke University | Canada |
| N=5 | Master’s students | | | - Laval University | Canada |
| N=5 | Research professionals | | | - Laval University | Canada |
