# Supplementary material for: How can continuing professional development better promote shared decision-making? Perspectives from an international collaboration
Source: Implement Sci. 2011 Jul 5;6:68. doi: 10.1186/1748-5908-6-68 (PMC3154854; doi:10.1186/1748-5908-6-68)
Supplement: Additional file 3 — Appendix 3. Questions and Answers for the Small Group Discussions [file 1748-5908-6-68-S3.DOC]

**Appendix 3: Questions and Answers for the Small Group Discussions**

|  | Question | Answers |
| --- | --- | --- |
| 1 | 1.1. Is anything missing from the environmental scan? How can we improve the scan? | - Improve methods - Find a balance between being more systematic (searching for more material, performed a Cochrane systematic review) and moving forward with existing material (were all usual physicians’ groups, nursing professionals’ organizations and allied health professionals’ groups contacted?) - Extract the following data: whether programs used existing structures (platforms), the types of learning activities practiced (skills, attitudes, or knowledge), providers’ perceptions of what they needed - Appraise programs using a set of accreditation standards to be developed from different countries’ standards - Perform subgroup analyses (e.g., country, targeted professional, clinical area, pre or post-licensure training program) - Determine best practices (construct an exemplary program) |
|  | 1.2. What are the key messages from the environmental scan and from each country’s presentation? | - Programs and their key success factors are poorly reported - There are many programs with great variety (duration, format, mode of delivery, content) heterogeneity - There is a need to evaluate the benefits of SDM training as a stand-alone program vs SDM training with another program - There is a need to determine set of core competencies in SDM - There is a need to determine criteria for certifying programs based on programs’ ability to meet SDM and accreditation standards |
| 2 | 2.1 At a minimum, what do you think should be included in a typical SDM-CPD program? Should a minimum set of competencies be covered? If yes, which competencies? | - Return to the definition of Makoul and Clayman’s integrated model (2006) to identify core competencies for SDM [37] |
|  | 2.2 What should the next grant proposal have as a research agenda? | - To establish an international consensus on core SDM competencies for SDM programs across health professions - To reach a consensus on the outcomes by which to evaluate CPD interventions, by determining optimum conditions (e.g., the duration of interventions, intervention strategies) - To develop an evaluation framework - To develop a grid for the accreditation of CPD-SDM programs - To build a CPD-SDM program - To develop a train-the-trainers program |
|  | 2.3 What are the next steps for this proposal? | - Member checking with the developers of the SDM-CPD programs covered in the environmental scan - Improving and finalising the scan - Mapping elements from existing programs, Delphi survey of professionals - Optimizing the environmental scan (see question 1) - Interviewing CME stakeholders on the following aspects (list not exhaustive): do they see value to an inventory of SDM training programs, how would they like this inventory be made available to them, is there anything else that could be added to the inventory. |
