# Supplementary material for: How can continuing professional development better promote shared decision-making? Perspectives from an international collaboration
Source: Implement Sci. 2011 Jul 5;6:68. doi: 10.1186/1748-5908-6-68 (PMC3154854; doi:10.1186/1748-5908-6-68)
Supplement: Additional file 4 — Appendix 4. Workshop Evaluation [file 1748-5908-6-68-S4.DOCX]

**Appendix 4: Workshop Evaluation**

|  | | Average score (1=Not at all, 5=Definitively) |
| --- | --- | --- |
| 1. Where objectives of this workshop made clear? | | 4.7 |
| 2. Did the speakers meet the objectives? | | 4.5 |
| 3. Were the speakers clear and effectives? | | 4.4 |
| 4. Were the discussion sessions useful? | | 4.8 |
| 5. Was the material relevant to your area of interest? | | 4.4 |
| 6. Were the presentations scientifically balanced, objective? | | 4.3 |
| 7. Was the content current? | | 4.7 |
| 8. Did the speakers challenge you to think about the topic in new ways? | | 4.7 |
| 9. Did the speakers and moderator encourage involvement of the audience? | | 4.8 |
| 10. Overall, how would you rate this workshop? | | 4.7 |
|  | | |
| Weaknesses / what needs improvement | - I did not see weaknesses, the critical point is that very few people had personal trainee experiences - I liked everything. - Give speakers more time to let them develop their argument. - The talk on countries’ representations might have been more directed so as to compare specific aspects between countries. - Lack of clarity re: aspect of collaboration and the environmental scan (not sure of meaning). - More background information about educational tools before the meeting could have been useful; more time should have been allotted for future plans (not just definitions). - Perhaps more preparatory work could help those with less background in the field. | |
| What you propose as the next step | - To reach a consensus on a training program and write a grant proposal for international studies. Thanks for this very kind initiative. - Questions to guide the interpretation of reviews and direct a future agenda and grant applications. - Follow up on the results of the extraction. I would like to continue to be part of CPD implementation. - Looking into patients' competencies and using a more participatory approach in research. - Presentation/overview of excerpt data in the first part of the program. - Identify and confirm activities for each component of SDM. - A discussion group to compare the views of students to those of senior researchers. - I would be interested in knowing how the objectives of SDM training could be assessed. | |
